# Cardiac Expression of Tnnt1 Requires the GATA4-FOG2 Transcription Complex

**DOI:** 10.1100/tsw.2009.75

**Published:** 2009-07-04

**Authors:** Nikolay L. Manuylov, Sergei G. Tevosian

**Affiliations:** ^1^ Department of Genetics, Dartmouth Medical School, Hanover, NH, USA; ^2^ Norris Cotton Cancer Center, Dartmouth Medical School, Hanover, NH, USA

**Keywords:** Heart, GATA4, FOG2, Zfpm2, Tnnt1

## Abstract

Previous work by us and others has shown that the loss of interaction between GATA4 and FOG2 protein partners is embryonic lethal due to heart failure at embryonic day (E) 13.5; however, the role of this important protein duo in various cardiac compartments (e.g., myocardial, endocardial, or epicardial cells) remains to be understood. Although a dual role (both as an activator and a repressor) for the GATA4-FOG2 transcriptional complex has been put forward, the specific genes under GATA4-FOG2 control in the developing heart have remained largely elusive. Since the myocardial-restricted *Fog2* re-expression in the *Fog2* null embryos is sufficient to extend their life span, identification of GATA4-FOG2 target genes in cardiomyocytes could shed light on the molecular mechanism of GATA4-FOG2 action in these cells. We report here that cardiac expression of slow skeletal troponin T (*Tnnt1*) strictly depends on the physical interaction between GATA4-FOG2 in the myocardium of both atria and ventricles.

### INTRODUCTION

The multitype, zinc-finger proteins of the FOG (Friend of GATA) family control biological activities of GATA-binding (GATA) transcription factors (for review, see [1,2]). The role for FOG2 (ZFPM2, Mouse Genome Informatics) protein in cardiac development has been firmly established. Initial characterization of the *Fog2* gene revealed prominent expression in several developing organ systems (e.g., brain, heart, and gonads)[3,4,5]. *Fog2*$^{-/-}$ (null) embryos die at mid-gestation (~E13.5), with a cardiac defect characterized by a thin ventricular myocardium, common atrioventricular (AV) canal, and the Tetralogy of Fallot malformation[6,7]. Importantly, *Fog2* gene loss affects the development of cardiac vasculature[7]. While the formation of an intact epicardial layer and expression of epicardium-specific genes in *Fog2* null mutants proceed apparently as normal, markers of cardiac vessel development are not detected[7,8]. Importantly, KDR expression is not detected in the epicardial layer of the *Fog2* knockout mice. KDR (FLK1, VEGFR2), the major receptor for VEGF (vascular endothelial growth factor), is an important marker of vascular cells and is absolutely essential for vascular development (e.g., [9], see [10] for a review).

This earlier work drew attention to the role that FOG2 and GATA4 play in the development of the cardiac vascular and epicardial cells. The early demise of the *Gata4* null embryos had limited the analysis of "*Gata4*-less" cardiac development to a narrow window between E7.0 and E9.0. Moreover, examination of the *Gata4* null mutants did not reveal a substantial down-regulation of any prospective GATA4 target genes and the cardiac manifestation of the knockout (cardia bifida) was attributed to a nonautonomous effect[11,12]. Additionally, as *Gata4*$^{-/-}$ ES cells could contribute to the developing heart and express a wide variety of cardiac markers[13], the significance of GATA4 expression in the cardiac compartment remained uncertain. Understanding the role for the GATA4-FOG2 complex (rather than for each protein separately) was facilitated by generating a *Gata4*$^{ki}$ line of mice; "ki" is a V217G mutation in GATA4 that specifically cripples the interaction between GATA4 and FOG proteins[14]. *Gata4*$^{ki/ki}$ mutants exhibited a similar (although not identical) phenotype to the *Fog2* null mutants[14]; this work reinforced the importance of the GATA4 protein and the GATA4-FOG2 interaction for both cardiogenesis, and the development of the cardiac vascular and epicardial cells. Re-examination of the cardiac defects in *Gata4* chimeric embryos[15], the recent generation of animals with cardiac-limited ablation of *Gata4* that have distinct cardiac-specific defects[16,17], as well as a report connecting mutations in the *GATA4* gene to congenital heart defects in humans[18], further confirmed the pivotal role for GATA4 in cardiogenesis.

The absence of the GATA4-FOG2 complex is embryonic lethal at E13.0–13.5 due to heart failure; however, the mechanism by which GATA4-FOG2 interaction loss translates into heart failure remains to be understood. Given that myocardial-restricted *Fog2* re-expression is sufficient to rescue cardiac vascular development and extend the life span of the *Fog2*-null embryos[7,19], we reasoned that GATA4-FOG2 target genes should exist in the myocardium. We have now identified the *skeletal troponin Tnnt1* gene as a myocardial target of the GATA4-FOG2 transcriptional complex. This finding was unexpected, since *Tnnt1* is mostly expressed in skeletal muscle where TNNT1 forms a part of the skeletal troponin-tropomyosin complex. Now our data demonstrate that cardiac expression of *Tnnt1* requires GATA4-FOG2 interaction.

### EXPERIMENTAL PROCEDURES

#### Animals

*Fog2* heterozygous and *Gata4*$^{ki}$ heterozygous animals were crossed to generate *Fog2*$^{-/-}$ and *Gata4*$^{ki/ki}$ (both mixed 129xC56BL/6 background); the generation and genotyping of *Fog2* transgenic, *Fog2*- and *Gata4*-targeted animals have been previously described[7,14]. *Mhc-Fog2* transgenic mice[7] were genotyped with primers specific for *Fog2* cDNA P3 5'-CAACTGCATTGTGTACAGC-3' and P8 5'-GCTCTTGGTGCATTGTGGGAAG-3'.

#### Affymetrix Microarray Analysis of Gene Expression

Embryonic hearts were dissected from E12.5 wild-type and *Gata4*$^{ki/ki}$ or *Fog2*$^{-/-}$ mutant embryos and transferred to RNAlater solution (Ambion). RNA was isolated with an RNeasy Mini Kit (Qiagen) by standard protocol and subsequently treated with DNAseI (Roche) to remove any possible DNA contamination. DNAseI was heat-inactivated for 15 min at 70$^{o}$C, and RNA was precipitated by standard protocol and diluted in 20 µl $H_2O$. Affymetrix oligonucleotide arrays were used for RNA expression analysis[20,21]. The array experiment was performed by the Dartmouth Genomic and Microarray Laboratory, according to the standard protocol. The microarray data have been deposited at the GEO database GSE14906 and were analyzed using Gene Traffic Software (Iobion Informatics).

### Transgenic Mice

A 5' 2.4-kb *Tnnt1* mouse genomic fragment was obtained by PCR using primers *tnnt1_2.4F* 5'-AAGTTTGAGGGCTGAGCCAT-3' and *tnnt1pR* GGCTGGGTCCACAGATGCTGTA; the conserved fifth intron of the mouse *Tnnt1* gene was similarly generated with primers *tnnt1iF* 5'-TTGAACTCATAGCAACTCTC-3' and *tnnt1_2.4R* 5'-TTAAGAGTTAAGGTTGGCTG-3'. To identify *cis*-regulatory elements responsible for cardiac and skeletal muscle expression of *Tnnt1*, we fused the 5' fragment upstream of an ATG codone of the *lacZ* reporter gene in pSDKlacZpA (a kind gift of Janet Rossant), while the intron sequence was subsequently inserted 3' to the LacZ-SV40 polyA sequences to generate pTnnt1-LacZpa. The fragment of pTnnt1-LacZpa containing the *Tnnt1* regulatory sequences directing expression from the LacZ-pA reporter was isolated using standard methods[22]. Transgenic animals were generated by the Dartmouth Transgenic and Genetic Construct Shared Resource Center by pronuclear injection into fertilized eggs. $F_0$ embryos were collected and analyzed at E12.5 by an X-gal staining assay.

### Whole Mount *In Situ* Hybridization

Embryos at various stages were removed from the uterus and their internal organs were removed to expose the heart; alternatively, whole embryonic hearts were dissected out. Embryonic tissues were fixed with 4% paraformaldehyde (PFA) in 1xPBS at 4°C overnight. Further processing of embryos and *in situ* hybridization analysis were carried out essentially as described[23]. *Tnnt1* and *Tnnt2* dig-labeled RNA probes have been generated by RT-PCR from E12.5 hearts. To generate the *Tnnt1* probe, we used primers *tnnt1pF* 5'-GGTCAAGGCAGAACAGAAGC-3' and *tnnt1pR* 5'-CTCCACACAGCAGGTCATGT-3'; *Tnnt2* probe primers are *tnnt2pF* 5'-CGGAAGAGTGGGAAGAGACA and *tnnt2pR* 5'-AGCTAAGCCAGCTCCCACTA-3'. Hearts were photographed and images were processed and assembled as previously described[22,24].

### Quantitative RT-PCR Analysis

The hearts (ventricles and atriums) were dissected in PBS from E12.5 or E17.5 embryos, and transferred in RNAlater solution (Ambion). RNA was isolated with an RNeasy Mini Kit (Qiagen) in 30 µl of RNAse-free TE buffer. During isolation, RNA samples were treated with RNAse-Free DNAse set for 15 min on RNeasy columns (Qiagen), according to the manufacturer's instructions. Each sample was divided into two aliquots, one of which was reverse transcribed using the M-MLV reverse transcriptase (Invitrogen), following the manufacturer's instructions. The second aliquot was used as a control without reverse transcription to identify and discard samples with DNA contamination. All real-time PCR assays were carried out using SYBR Green Universal PCR Master Mix (Applied Biosystems). The PCR reactions contained 25 ng of cDNA and gene-specific primers at a final concentration of 1 µ*M* each. The assays were run under standard SYBR Green conditions on the ABI 7500 instrument. A standard curve for each gene was generated using serial dilutions of cDNA. Relative expression levels for each sample were determined in the same run and were expressed as the ratio of the RNA amount (of interest) to the amount of control RNA (*Gapdh*). SYBR Green reactions were performed in duplicates and the experiments were repeated independently at least three times (for at least three samples). Gene-specific primers were designed using the Primer Express software (Perkin Elmer Life Sciences), namely:

Gapdh-qRT_F 5'-GCTCACTGGCATGGCCTTCCGTG-3';
Gapdh-qRT_R 5'-TGGAAGAGTGGGAGTTGCTGTTGA-3';
Tnnt1-qRT_F 5'-GGTCAAGGCAGAACAGAAGC-3'; and
Tnnt1-qRT_R 5'- GCGGTTGTAGAGCACATTGA-3'

### ß-Galactosidase Assay

Embryos were fixed and stained using X-gal essentially as previously described[22]. The staining was continuously monitored until a satisfactory color development was achieved (2–5 h). Embryos were then fixed overnight in 4% PFA in PBS and photographed as previously described[22].

## RESULTS

### Microarray Analysis of RNA Expression in FOG2 and GATA4 Mutant Hearts

In order to identify the targets of GATA4-FOG2 action in mammalian heart development, we performed Affymetrix microarray comparisons of gene expression in normal and mutant hearts at E12.5. We compared RNA samples from both *Fog2* null and *Gata4*$^{ki/ki}$ mutant E12.5 hearts to the wild-type control E12.5 hearts. We reasoned that as the phenotypes of the *Fog2* knockout and *Gata4*$^{ki/ki}$ mutation are similar[7,14], we should expect to identify a similar set of differentially expressed genes in both experiments. As an additional control, we expected to find the *Fog2* gene expression absent in the mutant (null) *Fog2* cardiac sample, but not *Gata4*$^{ki/ki}$ sample.

The microarray profiling yielded surprisingly few gene sets that were differentially represented (~2.5 times up- or down-regulated) in the mutant samples vs. controls. Importantly, the results were consistent between *"Fog2"* and *"Gata4*$^{ki}$*"* experiments (similar gene sets were recovered), with the exception of the gene set corresponding to the *Fog2* gene that was absent in the *Fog2* mutant sample, as we had predicted (Supplemental Table 1; see also [25]). The results of the microarray experiment are available at the GEO database (GSE14906).

### *Tnnt1* is a Target of the GATA4-FOG2 Transcription Complex

Microarray experiments have identified *Tnnt1* as a target of GATA4-FOG2 activation in the heart. Based on the microarray data, the expression of *Tnnt1* was down-regulated ~5 times in the *Fog2*-null sample and ~7 times in the *Gata*$^{ki/ki}$ sample; *Tnnt1* was the "most down-regulated" gene in both mutants (see also Supplementary Table in Smagulova et al.[25]). *Tnnt1* was also the only gene of the troponin group that was down-regulated in the *Gata4* and *Fog2* mutant hearts; other troponins (e.g., *Tnni1, Tnnc1*, or cardiac-restricted *Tnnt2* and *Tnni3*) were expressed at a similar level in control and mutant GATA4/FOG2 samples (not shown). Given this strong dependence of cardiac *Tnnt1* expression on the GATA4-FOG2 interaction, we decided to pursue the analysis of *Tnnt1* expression further.

### *Tnnt1* Expression in Cardiac Development

Expression of the *Tnnt1* gene in the rodent heart has been previously documented[26,27] and is consistent with our data. The whole-mount *in situ* hybridization (WISH) experiment using anti-*Tnnt1* RNA as a probe demonstrated that, at E9.5, the embryonic heart of a mouse is positive for *Tnnt1*, with expression visible in the outflow track and in the forming interventricular groove (Fig. 1A,B). At E10.5, *Tnnt1* expression expands posteriorly towards the apex; the expression also appears in the left ventricle (Fig. 1C–E). However, at E11.5, the expression in the outflow track is down-regulated and by E12, the expression is mostly confined to the ventral interventricular groove with some expression in the left ventricle; from about E12.0, the outflow track cells are negative for *Tnnt1* (Fig. 1F). In the E12.5 heart, interventricular *Tnnt1* expression expands laterally and by E14.5, the gene is expressed throughout the ventral side of the left ventricle; in the right ventricle, the expression is enhanced in the apical region, while the cells in the outflow

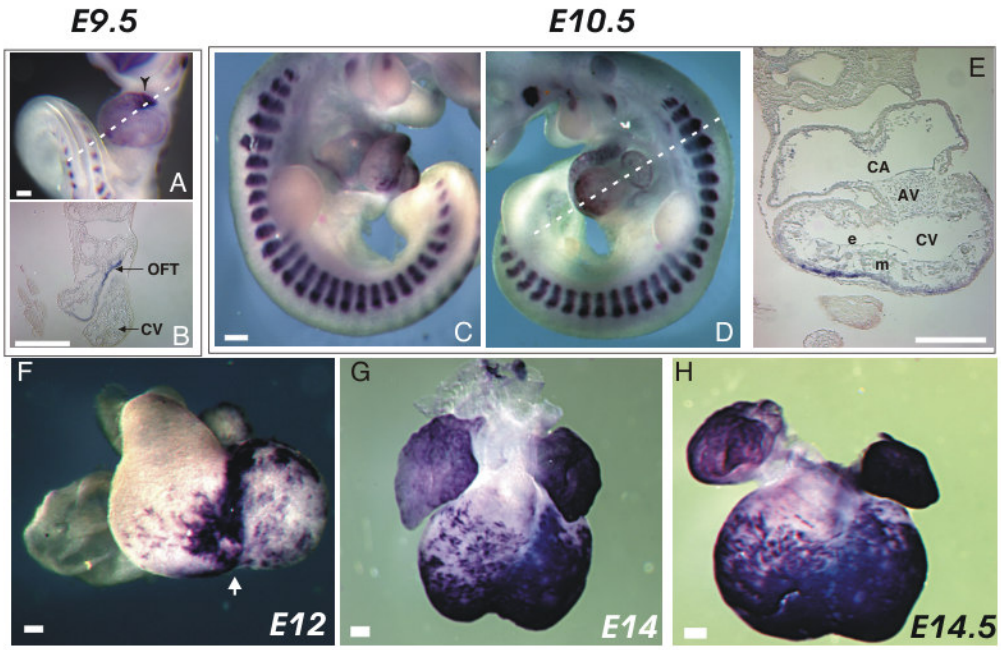

**FIGURE 1.** The dynamic expression of *Tnnt1* in the murine embryonic heart. WISH was performed with a *Tnnt1* antisense RNA probe on E9.5–10.5 embryos (A–E) or isolated hearts E12.0–14.5 (F–H). Whole embryo samples (A,D) were sectioned (B,E); the white dotted line in (A) and (D) indicates plane of sectioning. Note the expression in the outflow track at E9.5 (A, arrowhead) and in the interventricular groove (F, arrow). Scale bar, 100 µm (A,C,F–H) and 200 µm (B,E). AV, atrioventricular canal; CA, common atrium; CV, common ventricle; e, endocardium; m, myocardium, OFT, outflow tract.

track remain negative (Fig. 1G,H). The *Tnnt1* atrial expression becomes prominent at E12.5, with the left atrium being more positive.

### *Tnnt1* RNA Expression in *Fog2* Null and *Gata4*$^{ki/ki}$ Hearts

Microarray analysis and qRT-PCR both reveal a dramatic down-regulation of *Tnnt1* expression upon GATA4-FOG2 interaction loss. In accordance with microarray data, qRT-PCR demonstrated a significant down-regulation of *Tnnt1* in E12.5 mutant hearts (Fig. 2A). WISH corroborated this down-regulation in GATA4-FOG2 mutants (Fig. 2B–D). The residual expression in the *Fog2* null E12.5 heart (Fig. 2C) resembles the earlier (~E9.5) wild-type pattern, with positive cells persisting in the outflow track and the apical portion of the interventricular groove. No residual *Tnnt1* expression is apparent in the atria or ventricles of the *Gata4* mutant (*Gata4*$^{ki/ki}$) at E12.5 (Fig. 2D). Importantly, the noncardiac expression of *Tnnt1* (e.g., in skeletal muscle) remains intact in both *Fog2* and *Gata4* mutants (data not shown).

### *Tnnt1* Expression is Increased *in αMhc-Fog2* Transgenic Animals

To validate *Tnnt1* as a *bona fide* target of the GATA4-FOG2 complex, we performed additional experiments. *Fog2* expression is decreased in the developing heart shortly after E16.5[4]. *Tnnt1* expression was reported to follow a similar trend[26,27]. If the GATA4-FOG2 complex is required for *Tnnt1* activity, FOG2 concentration could be limiting and therefore responsible for *Tnnt1* down-regulation in the late gestation heart. In this case, cardiac *Fog2* overexpression should be sufficient for increasing *Tnnt1* levels in the heart. To test this possibility, we took advantage of the transgenic mice that

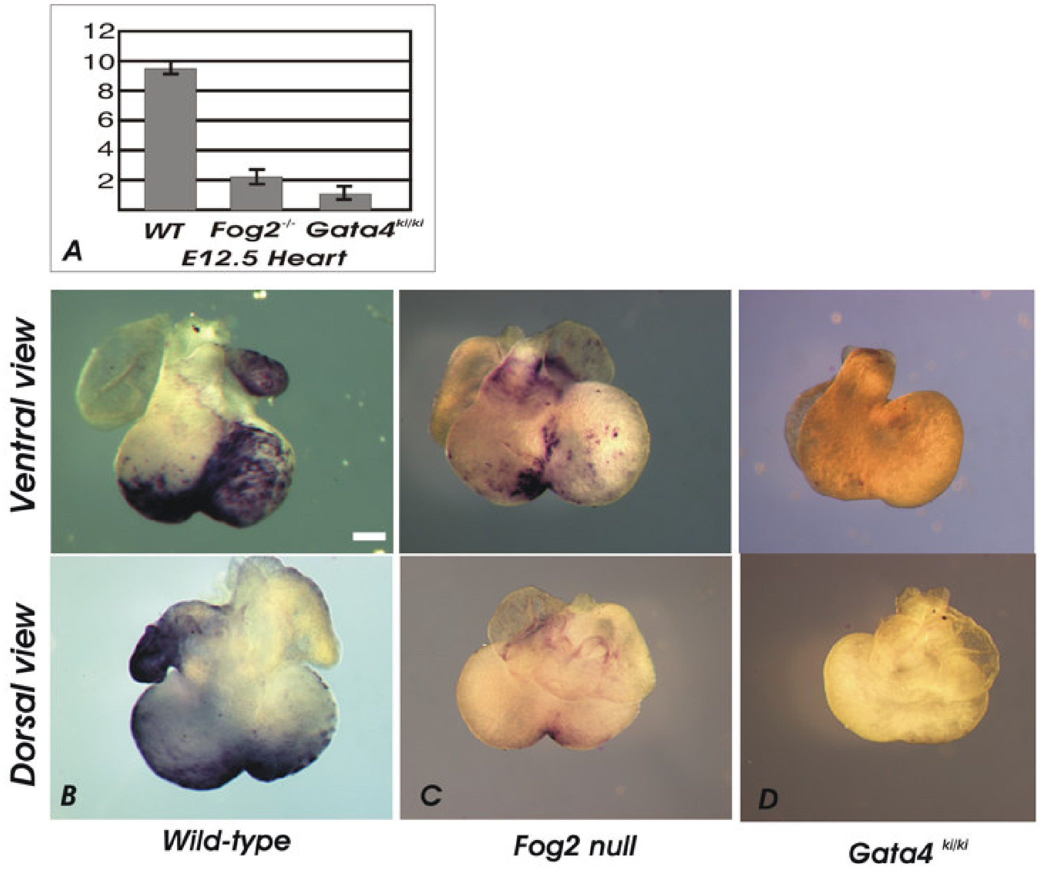

**FIGURE 2.** *Tnnt1* expression requires GATA4-FOG2 interaction. (A) Real-time PCR analysis of the *Tnnt1* gene expression in wild-type, *Fog2*$^{-/-}$, and *Gata4*$^{ki/ki}$ E12.5 hearts; the y axis shows values for both genes normalized to the *Gapdh* RNA copy number. (B–D) WISH was performed with a *Tnnt1* antisense RNA probe on isolated hearts from E12.5 control (B), *Fog2* null (C), and *Gata4*$^{ki/ki}$ (D) mutant embryos. Scale bar, 100 μm.

express *Fog2* under the control of regulatory sequences from the cardiac alpha myosin heavy chain (*αMhc*) promoter[7]. The *αMhc* promoter directs expression specifically to cardiomyocytes by E10.5[28]; the *αMhc-Fog2* transgenic animals have been previously described and were successfully used to rescue the lethality of *Fog2*$^{-/-}$ embryos at ~E14.5 from cardiac pathology[7,19].

As was shown previously[26,27], embryonic *Tnnt1* RNA expression is transient in the murine heart and starts to decline after E16.5. Accordingly, WISH with control E17.5 hearts showed low levels of cardiac *Tnnt1* expression (Fig. 3A). In contrast, in the E17.5 *αMhc-Fog2* transgenics, the *Tnnt1* expression level remains high in the atria, the known preferential site of *αMhc* expression at this stage (e.g., [29]) (Fig. 3B, arrows). qRT-PCR confirms that *Tnnt1* levels are elevated in the *αMhc-Fog2* neonatal animals compared to the controls (Fig. 3C). This demonstrates that elevating *Fog2* levels in cardiomyocytes is sufficient for increasing *Tnnt1* expression.

### *Tnnt1* Expression is Restored in Hearts with Myocardial-Restricted *Fog2*

As *Tnnt1* expression is increased in *αMhc-Fog2* transgenics, we sought to determine whether myocardial FOG2 is sufficient to recapitulate (rescue) *Tnnt1* cardiac expression in the otherwise *Fog2*-null fetuses. To test this, we crossed the *αMhc-Fog2* animals to *Fog2*$^{+/-}$ mice to generate *αMhc-Fog2;Fog2*$^{+/-}$ animals,

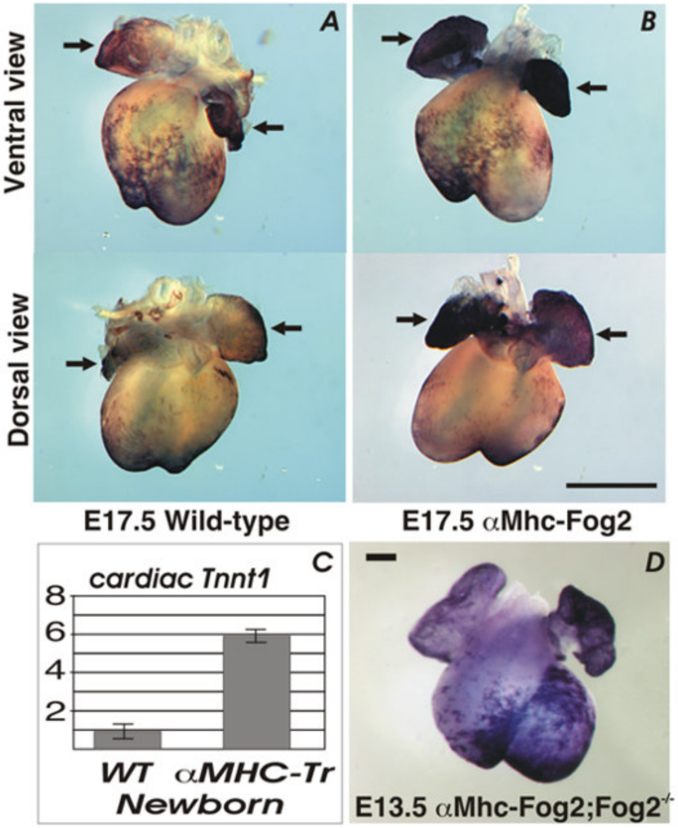

**FIGURE 3.** FOG2 regulates *Tnnt1* expression in myocardium. (A,B) WISH was performed with *Tnnt1* antisense RNA probe on isolated hearts from E17.5 control (A) and *αMhc-Fog2* transgenic embryos (B). Note enhanced expression in the atria of the transgenic embryos compared to the control (arrows). (C) Real-time PCR analysis of the *Tnnt1* gene expression in wild-type and *αMhc-Fog2* newborn hearts; the y axis shows values for both genes normalized to the *Gapdh* RNA copy number. (D) WISH was performed with *Tnnt1* antisense RNA probe on an isolated heart from E13.5 *αMhc-Fog2;Fog2*$^{-/-}$ mutant embryo; the expression pattern resembles that of the control heart (e.g., Fig. 2B) and not the *Fog2* null mutant (e.g., Fig. 2C). Scale bar, 1 mm (B) and 100 µm (D).

and backcrossed these to the *Fog2*$^{+/-}$ animals to obtain and examine the *αMhc-Fog2;Fog2*$^{-/-}$ fetuses. During mid-gestation, αMhc promoter directs expression to the ventricular myocardium[28]; hence, the *αMhc-Fog2/Fog2*$^{-/-}$ fetuses express *Fog2* cDNA driven by the *αMhc* promoter exclusively in the myocardium and are otherwise *Fog2* null. In the E13.5 hearts from these "rescued" embryos, the *Tnnt1* expression pattern is now restored (Fig. 3D) and appears indistinguishable from that in the contemporaneous wild-type hearts (compare Fig. 3D to Fig. 2B). We conclude that, although *Fog2* is expressed in all three cardiac layers, restoring FOG2 function specifically in the myocardium is sufficient to "rescue" *Tnnt1* expression.

### Proximal DNA Elements are Sufficient to Direct both Skeletal and Cardiac *Tnnt1* Expression

The *cis*-elements that are required to drive *Tnnt1* expression in skeletal muscle have not been defined[30]; even less is known about the transcriptional regulation of this gene in the heart. The genomic organization of the human and mouse *Tnnt1* gene has been reviewed[30]. The interesting feature of the *Tnnt1* gene is its location in very close proximity to cardiac-restricted troponin I (*Tnni3*); the distance between the *Tnnt1* and *Tnni3* is only 2.4 kb in the mouse (2.6 kb in the human)[30]. Intriguingly, the gene downstream of *Tnnt1* (14.7 kb; *Ppp1r12c*, *Mbs85*) is also highly expressed in the hearts of mice[31] and men[32]. However, both of these genetic neighbors are expressed normally in the *Gata4-Fog2* mutants, excluding the possibility of coregulation (not shown).

Inspection of the *Tnnt1* genomic locus using an ECR (evolutionary conserved region) browser[33] confirmed previously reported genomic organization of the locus[30]; however, outside of the TNNT1 coding sequence, we detected little conservation even between mammals, with the exception of the phylogenetically conserved fifth intron (Supplemental Fig. 1). This was unexpected, as a similar pattern and timing of cardiac expression was reported for a human and rat gene[34] and, hence, a better conservation of the regulatory sequences could have been expected. In order to identify the *cis*-regulatory elements that are responsible for cardiac-specific regulation of the *Tnnt1* gene by the GATA4-FOG2 complex, we generated a *LacZ* (bacterial β-galactosidase) fusion transgenic construct (Fig. 4A). The construct contained the 2.4-kb region upstream of the *Tnnt1* transcription start site with the 5' boundary delimited by the *Tnni3* ORF; we also inserted the phylogenetically conserved fifth intron 3’ to the LacZ-SV40polyA cassette to generate *2.4-Tnnt1-LacZpa-I5* (Fig. 4A). Transgenic construct was injected into fertilized eggs, and $F_0$ transgenic embryos were collected and analyzed at E12.5. The *2.4-Tnnt1-LacZpa-I5* sequences were fully sufficient to drive the expression of the *LacZ* reporter in the somites and developing skeletal muscle in all transgenic embryos (Fig. 4B). In addition to skeletal expression, we also observed cardiac-specific expression of the *lacZ* reporter in some (but not all) of these $F_0$ E12.5 embryos (Fig. 4C).

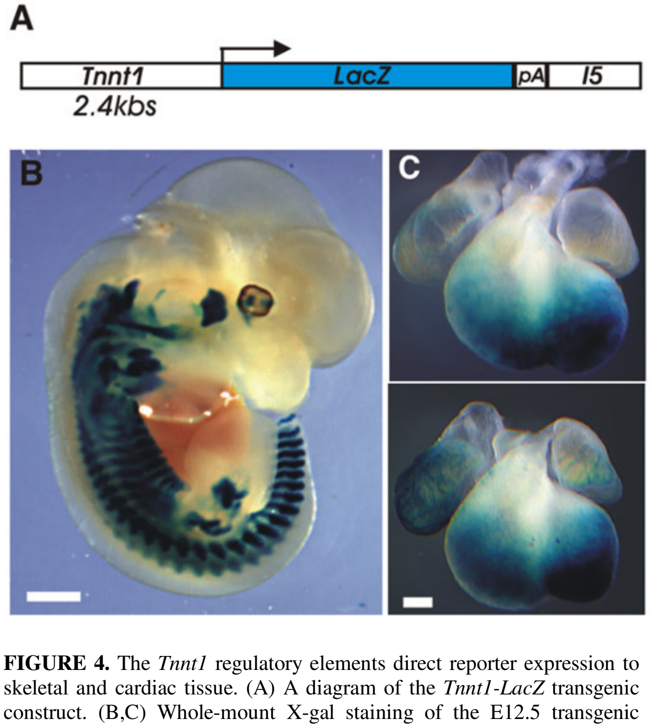

**FIGURE 4.** The *Tnnt1* regulatory elements direct reporter expression to skeletal and cardiac tissue. (A) A diagram of the *Tnnt1-LacZ* transgenic construct. (B,C) Whole-mount X-gal staining of the E12.5 transgenic embryo (B) and X-gal stained hearts from two different transgenic embryos (C). Compare the staining in (C) to the *Tnnt1* WISH-stained heart in Fig. 1. Scale bar, 200 µm (B) and 100 µm (C)

While it is possible that other transcription factors are responsible for *Tnnt1* regulation, we have not identified any myocardially expressed transcription factors in our microarray experiments. Hence, we reasoned that the GATA4-FOG2 complex is regulating *Tnnt1* expression directly through one of the GATA/TATC sequences. Despite numerous attempts, a chromatin immunoprecipitation (ChIP) assay from the E12.5 embryonic hearts did not pull down DNA containing GATA sites within *Tnnt1* DNA (not shown). Both antibodies used in this experiment (αGATA4 or αFOG2) were previously used successfully to isolate GATA-containing elements in the *Lhx9* regulatory region[25]. Experiments are currently in progress to address the mechanism of the GATA4-FOG2–dependent regulation of the *Tnnt1* by using the ES cell *in vitro* differentiation system.

### DISCUSSION

Interaction between GATA4 and FOG2 is required in normal cardiac development; however, the genetic mechanism of GATA4 and/or FOG2 action in the heart, and specifically in the myocardium, is not well understood. It has even been proposed that the myocardial defects in *Gata4* null hearts may be secondary to GATA4 loss in the proepicardium[15].

We now identify the *Tnnt1* gene as a target of the GATA4-FOG2 complex in the myocardium. The *cis*-acting elements that are required to drive this gene's expression in skeletal muscle have not been defined[30]; we now show that the 2.4 kb and sequences from intron 5 are sufficient to direct muscle-specific expression during mouse embryonic development. While all transgenic embryos express the transgene in the skeletal muscle, cardiac expression was observed in two out of five embryos, suggesting that while additional elements are required for consistent expression in the heart, sequences necessary for *Tnnt1* cardiac expression are present within the transgene's regulatory elements. Despite our repeated attempts, the cardiac ChIP assay could not detect a GATA4-FOG2 complex bound to GATA/TATC elements within these regulatory sequences. In light of these negative results, we conclude that *Tnnt1* is unlikely to be directly regulated by the GATA4-FOG2 complex in the heart.

A *2.4Tnnt1-LacZpA-I5* reporter we have generated is robustly expressed in embryonic skeletal muscle, thus indicating that the 2.4-kb fragment and intron 5 contain all the elements necessary for skeletal muscle expression. As skeletal muscle does not express FOG molecules, *Tnnt1* expression in this tissue has to be independent of GATA-FOG interaction; correspondingly, *Tnnt1* is expressed normally in skeletal muscle of the *Fog2* null and *Gata4*$^{ki/ki}$ mutants. Outside of skeletal muscle, *Tnnt1* RNA has been detected in several other tissues; the significance of this extraskeletal *Tnnt1* gene expression and its transcriptional regulation are not understood. In the murine and human heart, *Tnnt1* RNA expression was previously described; this expression is transient during embryogenesis and starts to decline in mice after E16.5[26,27]. Expression of *TNNT1* was also reported in several examined human embryonic stem cell lines (where it is lost upon differentiation[35]) and in aging hearts[36,37]. Furthermore, *Tnnt1* expression is dramatically induced in brains (neurons) of mice treated with ketamine[38]. Expression of *Tnnt1* outside of muscle tissue hints at alternative function other than its conventional structural role in the sarcomere.

In humans, intact TNNT1 in skeletal musculature is required to support life: a nonsense mutation in *TNNT1* causes an autosomal-recessive Amish Nemaline Myopathy (ANM). The children affected by ANM die of respiratory insufficiency, usually in their second year of life. The mutation (a stop codon) in exon 11 results in a deletion of the last 83 amino acids of the protein, removing the protein-binding modules that are necessary for TNNT1's structural function[39]. Although congestive heart failure has been commonly observed for the ANM patients, no evidence of primary cardiac involvement was reported[39].

While elucidating the function of *Tnnt1* (sTnT) in mammalian development and cardiogenesis will have to await this gene's deletion in mice, targeted disruption of its cardiac homologue *Tnnt2* (cTnT) is embryonic lethal at around E10[40,41]. It has been reported that, while nearly lacking a heartbeat, a minor twitching consisting of a few cardiomyocytes was observed in all of E10 and about half of E9 *cTnT*$^{-/-}$ (*Tnnt2*$^{-/-}$) embryos, suggesting that some developmentally regulated mechanism compensated partially for the lack of TNNT2[41]. Interestingly, the beating cells were observed in the outflow tract in these embryos, which is the zone of *Tnnt1* expression (Fig. 2A), in line with the author's hypothesis that *Tnnt1* is able to compensate for *Tnnt2* at this stage.

## SUPPLEMENTARY MATERIAL

### SUPPLEMENTAL TABLE 1
## Differentially Expressed Genes in E12.5 Control vs. *Gata4*$^{ki/ki}$ Hearts

| Gene Order | Affymetrix Probe | Fold Change | Sequence Accession Number | Gene Symbol | Gene Name |
|---|---|---|---|---|---|
| Downregulated genes | | | | | |
| 1. | 1426561_a_at | –5.81 | NM_001029836.1 | *Npnt* | Nephronectin |
| 2. | 1419606_a_at | –5.77 | NM_011618.1 | *Tnnt1* | Troponin T1 |
| 3. | 1428549_at | –5.48 | NM_028804.1 | *Ccdc3* | Coiled-coil domain containing 3 |
| 4. | 1417023_a_at | –5.3 | NM_024406.2 | *Fabp4* | Fatty acid binding protein 4, adipocyte |
| 5. | 1421144_at | –5.05 | NM_023879.2 | *Rpgrip1* | Retinitis pigmentosa GTPase regulator interacting protein 1 |
| 6. | 1452088_at | –4.15 | NM_028106.2 | *Zbed3* | Zinc finger, BED domain containing 3 |
| 7. | 1427457_a_at | –4.13 | NM_009755.2 | *Bmp1* | Bone morphogenetic protein 1 |
| 8. | 1423062_at | –4.08 | NM_008343.2 | *Igfbp3* | Insulin-like growth factor binding protein 3 |
| 9. | 1416515_at | –3.97 | NM_007984.2 | *Fscn1* | Fascin homolog 1, actin bundling protein (*Strongylocentrotus purpuratus*) |
| 10. | 1427476_a_at | –3.96 | NM_053084.1 | *Trim32* | Tripartite motif protein 32 |
| 11. | 1417860_a_at | –3.95 | NM_133903.2 | *Spon2* | Spondin 2, extracellular matrix protein |
| 12. | 1460232_s_at | –3.9 | NM_013821.3 | *Hsd3b2 /// Hsd3b6* | Hydroxysteroid dehydrogenase-2, delta<5>-3-beta /hydroxysteroid dehydrogenase-6, delta<5>-3-beta 1 |
| 13. | 1436399_s_at | –3.89 | NM_013724.2 | *Nrk* | Nik related kinase |
| 14. | 1417888_at | –3.81 | NM_023233.2 | *Trim13* | Tripartite motif protein 13 |
| 15. | 1427385_s_at | –3.78 | NM_011501.1 | *Strm* | Striamin |
| 16. | 1416033_at | –3.67 | NM_134142.1 | *Tmem109* | Transmembrane protein 109 |
| 17. | 1449319_at | –3.66 | NM_138683.2 | *Rspondin* | Thrombospondin type 1 domain containing gene |
| 18. | 1448147_at | –3.63 | NM_013869.4 | *Tnfrsf19* | Tumor necrosis factor receptor superfamily, member 19 |
| 19. | 1418908_at | –3.62 | NM_013626.3 | *Pam* | Peptidylglycine alpha-amidating monooxygenase |
| 20. | 1451331_at | –3.46 | NM_144828.1 | *Ppp1r1b* | Protein phosphatase 1, regulatory (inhibitor) subunit 1B |
| 21. | 1418723_at | –3.35 | NM_022983.3 | *Edg7* | Endothelial differentiation, lysophosphatidic acid G protein–coupled receptor 7 |
| 22. | 1438936_s_at | –3.35 | NM_009640.3 | *Ang1* | Angiopoietin 1 |
| 23. | 1418535_at | –3.32 | NM_016846.3 | *Rgl1* | Ral guanine nucleotide dissociation stimulator,-like 1 |
| 24. | 1451203_at | –3.28 | NM_013593.2 | *Mb* | Myoglobin |
| 25. | 1437733_at | –3.25 | NM_010124.2 | *Eif4ebp2* | Eukaryotic translation initiation factor 4E binding protein 2 |

*Table continues*

### SUPPLEMENTAL TABLE 1 (continued)

| Gene Order | Affymetrix Probe | Fold Change | Sequence Accession Number | Gene Symbol | Gene Name |
|---|---|---|---|---|---|
| Upregulated genes | | | | | |
| 1. | 1454866_s_at | 59.81 | NM_172469.3 | *Clic6* | Chloride intracellular channel 6 |
| 2. | 1433930_at | 43.92 | NM_152803.4 | *Hpse* | Heparanase |
| 3. | 1416645_a_at | 39.08 | NM_007423.4 | *Afp* | Alpha fetoprotein |
| 4. | 1434165_at | 22.67 | NM_172469.3 | *Clic6* | Chloride intracellular channel 6 |
| 5. | 1416646_at | 13.07 | NM_007423.4 | *Afp* | Alpha fetoprotein |
| 6. | 1427119_at | 11.21 | NM_011463.2 | *Spink4* | Serine protease inhibitor, Kazal type 4 |
| 7. | 1416468_at | 8.5 | NM_013467.3 | *Aldh1a1* | Aldehyde dehydrogenase family 1, subfamily A1 |
| 8. | 1418199_at | 6.92 | NM_053149.2 | *Hemgn* | Hemogen |
| 9. | 1460214_at | 5.9 | NM_008791.2 | *Pcp4* | Purkinje cell protein 4 |
| 10. | 1420664_s_at | 5.47 | NM_011171.1 | *Procr* | Protein C receptor, endothelial |
| 11. | 1422836_at | 5.36 | NM_134163.4 | *Mbnl3* | Muscleblind-like 3 (Drosophila) |
| 12. | 1423691_x_at | 5.32 | NM_031170.2 | *Krt2-8* | Keratin complex 2, basic, gene 8 |
| 13. | 1420647_a_at | 5.19 | NM_031170.2 | *Krt2-8* | Keratin complex 2, basic, gene 8 |
| 14. | 1429159_at | 4.93 | AK014514.1 | *4631408O11Rik* | RIKEN cDNA 4631408O11 gene |
| 15. | 1449169_at | 4.84 | NM_008216.3 | *Has2* | Hyaluronan synthase 2 |
| 16. | 1427428_at | 4.08 | NM_029465.3 | *Clec4g* | C-type lectin domain family 4, member g |
| 17. | 1435989_x_at | 4.02 | NM_031170.2 | *Krt2-8* | Keratin complex 2, basic, gene 8 |
| 18. | 1428942_at | 3.83 | NM_008630.2 | *Mt2* | Metallothionein 2 |
| 19. | 1418678_at | 3.69 | NM_008216.3 | *Has2* | Hyaluronan synthase 2 |
| 20. | 1456014_s_at | 3.69 | NM_153795.1 | *Fermt3* | Fermitin family homolog 3 (Drosophila) |
| 21. | 1423429_at | 3.59 | NM_008818.2 | *Rhox5* | Reproductive homeobox 5 |
| 22. | 1429146_at | 3.54 | NM_001160345.1 | *Svip* | Small VCP/p97-interacting protein |
| 23. | 1455599_at | 3.5 | NM_001033399.4 | *Gfod1* | Glucose-fructose oxidoreductase domain containing 1 |
| 24. | 1449425_at | 3.47 | NM_023653.4 | *Wnt2* | Wingless-related MMTV integration site 2 |
| 25. | 1427183_at | 3.45 | NM_146015.2 | *Efemp1* | Epidermal growth factor-containing fibulin-like extracellular matrix protein 1 |

Calculated by the Gene traffic program.

```
Mouse            1 -----GCCAAGCCTCTCTCTACCCAACTCTCTCTAATTATCTTTTTGTATTCCCCCATTGCCACCAG 62
Rat              1 -----GCCAAGCCTCTCTCTACTCAACTCTCACTAATTATCTTTTTGTATTCCCCTATTGCCACCAG 62
Human            1 -----GCCTAGCCCCTCTCCTCTAAACTCTTGCTAATTATCTCTTTTTAT-CCCCCACCACCAACAG 61
Pan_Trogloditus  1 -----GCCTAGCCCCTCTCCTCTAAACTCTTGCTAATTATCTCTTTTTAT-CCCCCACCACCAACAG 61
Macaca           1 -------------------------AACTCTTGCTAATTATCTCTTTGTAT-CCCCCACCACCAACAG 43
Bos_tauris       1 AAATGTCATGCCCACCCACCCACGAACTCTTGCTAATTATCTCTTTGTATCCCTCT-----CACCAG 63
```

**SUPPLEMENTAL FIGURE 1.** The alignment of the phylogenetically conserved *Tnnt1* intron5 sequences from six mammalian species; the position of the conserved TATC/GATA element is underlined.

### ACKNOWLEDGMENTS

The authors would like to thank the Dartmouth Transgenic and Genetic Construct Shared Resource Center for their help in generating the transient transgenic animals; we also want to thank Ms. Shawna Fox for proofreading. This work was supported in part by the NIH (R01HD42751, NICHD) award to SGT.

## References

1. Cantor, A.B. and Orkin, S.H. (2005) Coregulation of GATA factors by the Friend of GATA (FOG) family of multitype zinc finger proteins. *Semin. Cell Dev. Biol.* **16,** 117–128.
2. Sorrentino, R.P., Gajewski, K.M., and Schulz, R.A. (2005) GATA factors in Drosophila heart and blood cell development. *Semin. Cell Dev. Biol.* **16,** 107–116.
3. Lu, J.R., McKinsey, T.A., Xu, H., Wang, D.Z., Richardson, J.A., and Olson, E.N. (1999) FOG-2, a heart- and brain-enriched cofactor for GATA transcription factors. *Mol. Cell Biol.* **19,** 4495–4502.
4. Svensson, E.C., Tufts, R.L., Polk, C.E., and Leiden, J.M. (1999) Molecular cloning of FOG-2: a modulator of transcription factor GATA-4 in cardiomyocytes. *Proc. Natl. Acad. Sci. U. S. A.* **96,** 956–961.
5. Tevosian, S.G., Deconinck, A.E., Cantor, A.B., Rieff, H.I., Fujiwara, Y., Corfas, G., and Orkin, S.H. (1999) FOG-2: a novel GATA-family cofactor related to multitype zinc-finger proteins Friend of GATA-1 and U-shaped. *Proc. Natl. Acad. Sci. U. S. A.* **96,** 950–955.
6. Svensson, E.C., Huggins, G.S., Lin, H., Clendenin, C., Jiang, F., Tufts, R., Dardik, F.B., and Leiden, J.M. (2000) A syndrome of tricuspid atresia in mice with a targeted mutation of the gene encoding Fog-2. *Nat. Genet.* **25,** 353–356.
7. Tevosian, S.G., Deconinck, A.E., Tanaka, M., Schinke, M., Litovsky, S.H., Izumo, S., Fujiwara, Y., and Orkin, S.H. (2000) FOG-2, a cofactor for GATA transcription factors, is essential for heart morphogenesis and development of coronary vessels from epicardium. *Cell* **101,** 729–739.
8. Novak, K. (2000) Lost in the FOG. *Nat. Med.* **6,** 864.
9. Shalaby, F., Rossant, J., Yamaguchi, T.P., Breitman, M.L., and Schuh, A.C. (1995) Failure of blood island formation and vasculogenesis in flk-1 deficient mice. *Nature* **376,** 62–66.
10. Bautch, V.L. and Ambler, C.A. (2004) Assembly and patterning of vertebrate blood vessels. *Trends Cardiovasc. Med.* **14,** 138–143.
11. Kuo, C.T., Morrisey, E.E., Anandappa, R., Sigrist, K., Lu, M.M., Parmacek, M.S., Soudais, C., and Leiden, J.M. (1997) GATA4 transcription factor is required for ventral morphogenesis and heart tube formation. *Genes Dev.* **11,** 1048–1060.
12. Molkentin, J.D., Lin, Q., Duncan, S.A., and Olson, E.N. (1997) Requirement of the transcription factor GATA4 for heart tube formation and ventral morphogenesis. *Genes Dev.* **11,** 1061–1072.
13. Narita, N., Bielinska, M., and Wilson, D.B. (1997) Cardiomyocyte differentiation by GATA-4-deficient embryonic stem cells. *Development* **124,** 3755–3764.
14. Crispino, J.D., Lodish, M.B., Thurberg, B.L., Litovsky, S.H., Collins, T., Molkentin, J.D., and Orkin, S.H. (2001) Proper coronary vascular development and heart morphogenesis depend on interaction of GATA-4 with FOG cofactors. *Genes Dev.* **15,** 839–844.
15. Watt, A.J., Battle, M.A., Li, J., and Duncan, S.A. (2004) GATA4 is essential for formation of the proepicardium and regulates cardiogenesis. *Proc. Natl. Acad. Sci. U. S. A.* **101,** 12573–12578.
16. Oka, T., Maillet, M., Watt, A.J., Schwartz, R.J., Aronow, B.J., Duncan, S.A., and Molkentin, J.D. (2006) Cardiac-specific deletion of Gata4 reveals its requirement for hypertrophy, compensation, and myocyte viability. *Circ. Res.* **98,** 837–845.
17. Zeisberg, E.M., Ma, Q., Juraszek, A.L., Moses, K., Schwartz, R.J., Izumo, S., and Pu, W.T. (2005) Morphogenesis of the right ventricle requires myocardial expression of Gata4. *J. Clin. Invest.* **115,** 1522–1531.
18. Garg, V., Kathiriya, I.S., Barnes, R., Schluterman, M.K., King, I.N., Butler, C.A., Rothrock, C.R., Eapen, R.S., Hirayama-Yamada, K., Joo, K., Matsuoka, R., Cohen, J.C., and Srivastava, D. (2003) GATA4 mutations cause human congenital heart defects and reveal an interaction with TBX5. *Nature* **6,** 6.
19. Tevosian, S.G., Albrecht, K.H., Crispino, J.D., Fujiwara, Y., Eicher, E.M., and Orkin, S.H. (2002) Gonadal differentiation, sex determination and normal Sry expression in mice require direct interaction between transcription partners GATA4 and FOG2. *Development* **129,** 4627–4634.
20. Chee, M., Yang, R., Hubbell, E., Berno, A., Huang, X.C., Stern, D., Winkler, J., Lockhart, D.J., Morris, M.S., and Fodor, S.P. (1996) Accessing genetic information with high-density DNA arrays. *Science* **274**, 610–614.
21. Lipshutz, R.J., Fodor, S.P., Gingeras, T.R., and Lockhart, D.J. (1999) High density synthetic oligonucleotide arrays. *Nat. Genet.* **21,** 20–24.
22. Adameyko, I.I., Mudry, R.E., Houston-Cummings, N.R., Veselov, A.P., Gregorio, C.C., and Tevosian, S.G. (2005) Expression and regulation of mouse SERDIN1, a highly conserved cardiac-specific leucine-rich repeat protein. *Dev.*

*Dyn*. **233,** 540–552.
23. Wilkinson, D.G. (1992) *In Situ Hybridization*. IRL Press, Oxford.
24. Manuylov, N.L., Manuylova, E., Avdoshina, V., and Tevosian, S. (2008) Serdin1/Lrrc10 is dispensable for mouse development. *Genesis* **46,** 441–446.
25. Smagulova, F.O., Manuylov, N.L., Leach, L.L., and Tevosian, S.G. (2008) GATA4/FOG2 transcriptional complex regulates Lhx9 gene expression in murine heart development. *BMC Dev. Biol*. **8**, 67.
26. Krishan, K., Morgan, M.J., Zhao, W., and Dhoot, G.K. (2000) Slow troponin T mRNA in striated muscles is expressed in both cell type and developmental stage specific manner. *J. Muscle Res. Cell Motil*. **21,** 527–536.
27. Wang, Q., Reiter, R.S., Huang, Q.Q., Jin, J.P., and Lin, J.J. (2001) Comparative studies on the expression patterns of three troponin T genes during mouse development. *Anat. Rec.* **263,** 72–84.
28. Subbarayan, V., Mark, M., Messadeq, N., Rustin, P., Chambon, P., and Kastner, P. (2000) RXRalpha overexpression in cardiomyocytes causes dilated cardiomyopathy but fails to rescue myocardial hypoplasia in RXRalpha-null fetuses. *J. Clin. Invest*. **105,** 387–394.
29. Palermo, J., Gulick, J., Colbert, M., Fewell, J., and Robbins, J. (1996) Transgenic remodeling of the contractile apparatus in the mammalian heart. *Circ. Res*. **78,** 504–509.
30. Cullen, M.E., Dellow, K.A., and Barton, P.J. (2004) Structure and regulation of human troponin genes. *Mol. Cell. Biochem*. **263,** 81–90.
31. Dutheil, N., Yoon-Robarts, M., Ward, P., Henckaerts, E., Skrabanek, L., Berns, K.I., Campagne, F., and Linden, R.M. (2004) Characterization of the mouse adeno-associated virus AAVS1 ortholog. *J. Virol*. **78,** 8917–8921.
32. Tan, I., Ng, C.H., Lim, L., and Leung, T. (2001) Phosphorylation of a novel myosin binding subunit of protein phosphatase 1 reveals a conserved mechanism in the regulation of actin cytoskeleton. *J. Biol. Chem*. **276,** 21209–21216.
33. Ovcharenko, I., Nobrega, M.A., Loots, G.G., and Stubbs, L. (2004) ECR Browser: a tool for visualizing and accessing data from comparisons of multiple vertebrate genomes. *Nucleic Acids Res.* **32,** W280–286.
34. Barton, P.J., Felkin, L.E., Koban, M.U., Cullen, M.E., Brand, N.J., and Dhoot, G.K. (2004) The slow skeletal muscle troponin T gene is expressed in developing and diseased human heart. *Mol. Cell. Biochem*. **263,** 91–97.
35. Bhattacharya, B., Miura, T., Brandenberger, R., Mejido, J., Luo, Y., Yang, A.X., Joshi, B.H., Ginis, I., Thies, R.S., Amit, M., Lyons, I., Condie, B.G., Itskovitz-Eldor, J., Rao, M.S., and Puri, R.K. (2004) Gene expression in human embryonic stem cell lines: unique molecular signature. *Blood* **103,** 2956–2964.
36. Butte, A.J. and Kohane, I.S. (2006) Creation and implications of a phenome-genome network. *Nat. Biotechnol*. **24,** 55–62.
37. Lee, C.K., Allison, D.B., Brand, J., Weindruch, R., and Prolla, T.A. (2002) Transcriptional profiles associated with aging and middle age-onset caloric restriction in mouse hearts. *Proc. Natl. Acad. Sci. U. S. A.* **99,** 14988–14993.
38. Lowe, X.R., Lu, X., Marchetti, F., and Wyrobek, A.J. (2007) The expression of Troponin T1 gene is induced by ketamine in adult mouse brain. *Brain Res*. **1174,** 7–17.
39. Johnston, J.J., Kelley, R.I., Crawford, T.O., Morton, D.H., Agarwala, R., Koch, T., Schaffer, A.A., Francomano, C.A., and Biesecker, L.G. (2000) A novel nemaline myopathy in the Amish caused by a mutation in troponin T1. *Am. J. Hum. Genet*. **67,** 814–821.
40. Ahmad, F., Banerjee, S.K., Lage, M.L., Huang, X.N., Smith, S.H., Saba, S., Rager, J., Conner, D.A., Janczewski, A.M., Tobita, K., Tinney, J.P., Moskowitz, I.P., Perez-Atayde, A.R., Keller, B.B., Mathier, M.A., Shroff, S.G., Seidman, C.E., and Seidman, J.G. (2008) The role of cardiac troponin T quantity and function in cardiac development and dilated cardiomyopathy. *PLoS ONE* **3,** e2642.
41. Nishii, K., Morimoto, S., Minakami, R., Miyano, Y., Hashizume, K., Ohta, M., Zhan, D.Y., Lu, Q.W., and Shibata, Y. (2008) Targeted disruption of the cardiac troponin T gene causes sarcomere disassembly and defects in heartbeat within the early mouse embryo. *Dev. Biol*. **322,** 65–73.
